# Supplementary material for: Modified long-axis in-plane ultrasound technique versus conventional palpation technique for radial arterial cannulation: A prospective randomized controlled trial
Source: Medicine (Baltimore). 2020 Jan 10;99(2):e18747. doi: 10.1097/MD.0000000000018747 (PMC6959944; doi:10.1097/MD.0000000000018747)
Supplement: Supplemental Digital Content [file medi-99-e18747-s001.doc]

Supplemental table 1

Table. Complications Recorded During Arterial Cannulation

| Complications | M-LAINUT  (N =143)  n (%) | C-PT  (N = 142)  n (%) | P | OR (95% CI) |
| --- | --- | --- | --- | --- |
| Thrombosis | 0 | 0 |  |  |
| Hematoma | 4 (2.80) | 28 (19.72) | <0.001 | 8.54 (2.9 to 25.0) |
| Vasospasm | 0 | 0 |  |  |
| Edema | 0 | 0 |  |  |

The P values and 95% CIs are calculated from the χ^2^ test.
