# Supplementary figures and images for: Modified long-axis in-plane ultrasound technique versus conventional palpation technique for radial arterial cannulation: A prospective randomized controlled trial
Source: Medicine (Baltimore). 2020 Jan 10;99(2):e18747. doi: 10.1097/MD.0000000000018747 (PMC6959944; doi:10.1097/MD.0000000000018747)

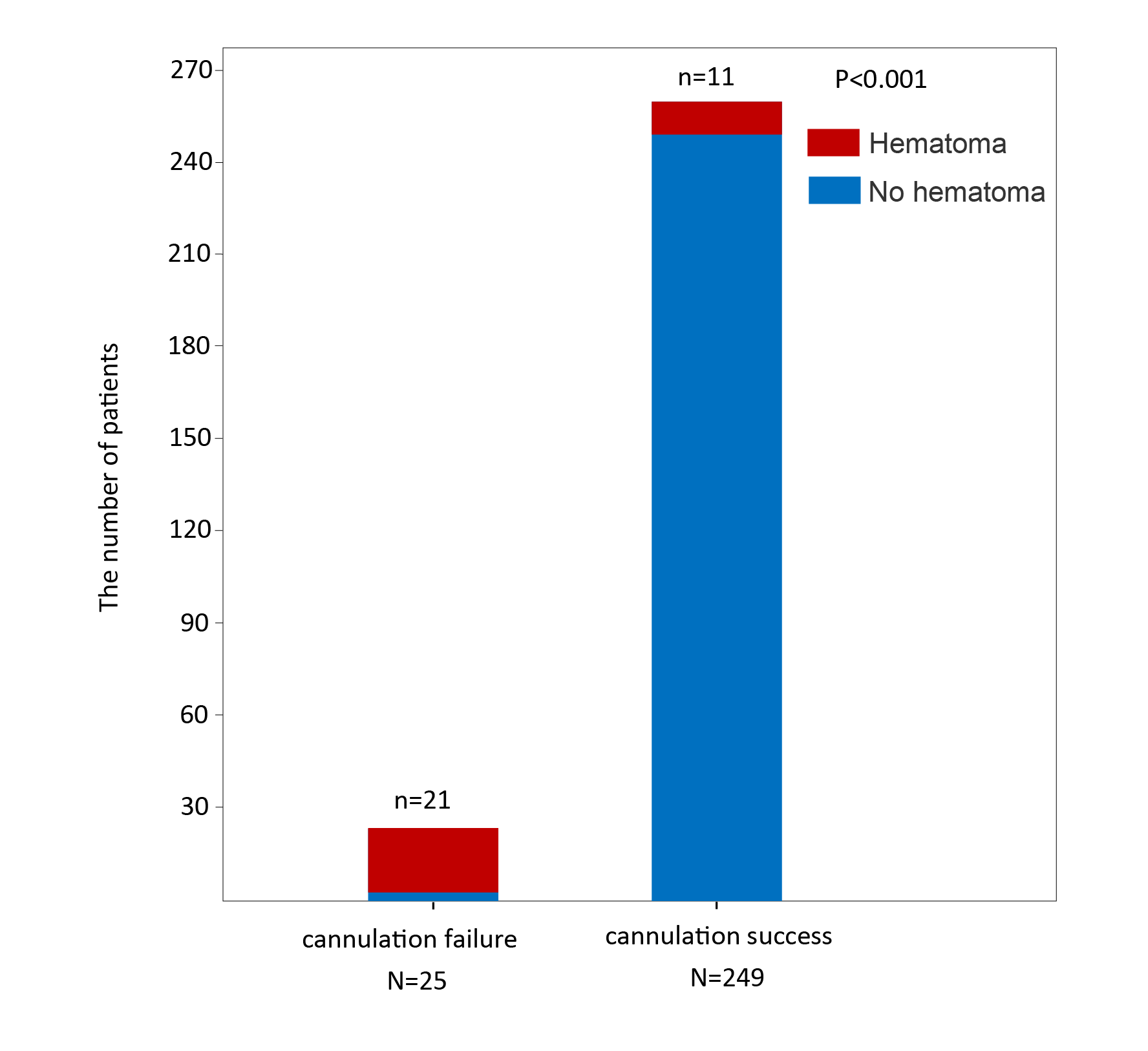

Supplement: Supplemental Digital Content [file medi-99-e18747-s002.tif]

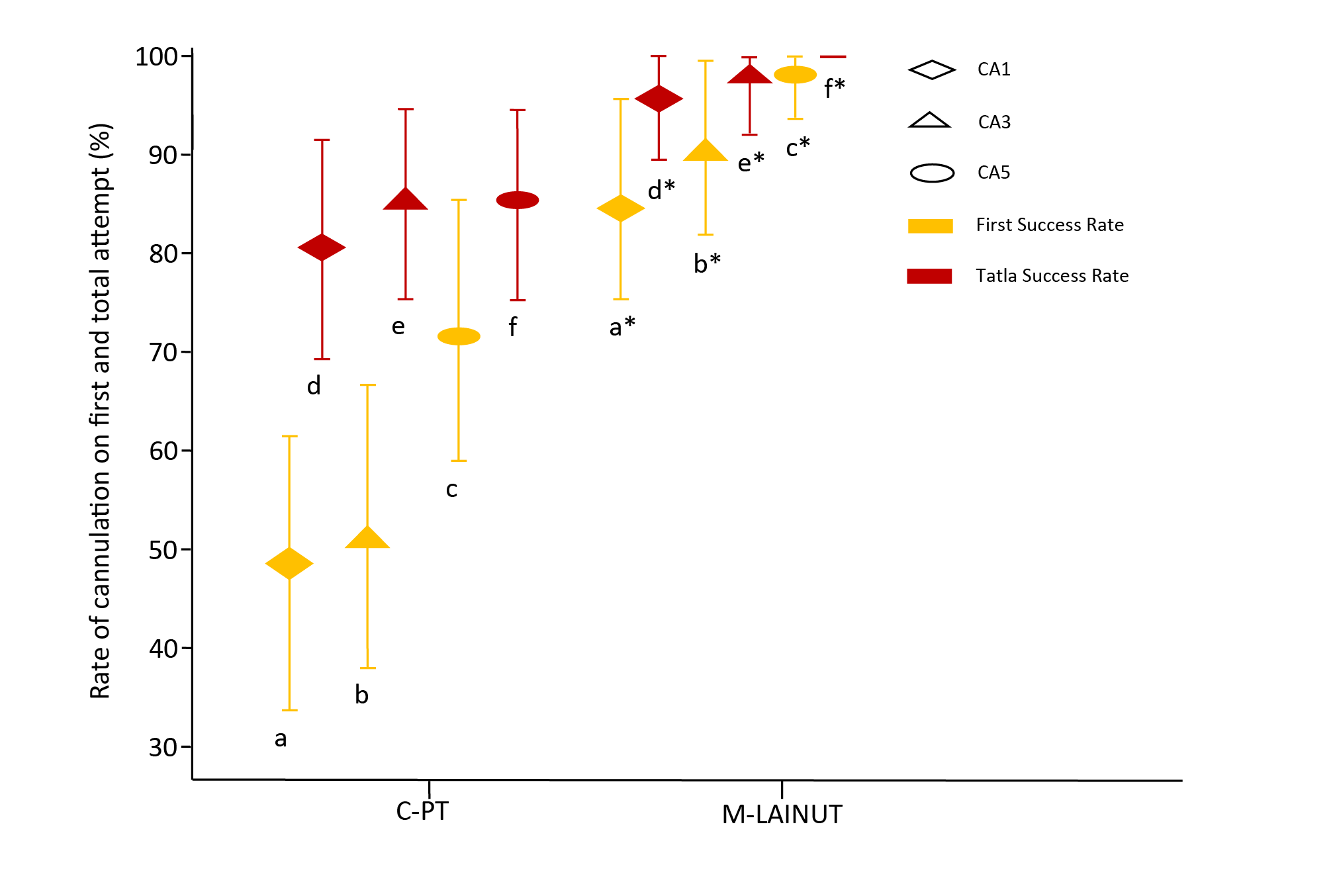

Supplement: Supplemental Digital Content [file medi-99-e18747-s003.tif]
